# The CCR4-NOT Complex Physically and Functionally Interacts with TRAMP and the Nuclear Exosome

**DOI:** 10.1371/journal.pone.0006760

**Published:** 2009-08-25

**Authors:** Nowel Azzouz, Olesya O. Panasenko, Geoffroy Colau, Martine A. Collart

**Affiliations:** Department of Microbiology and Molecular Medicine, Faculty of Medicine, University of Geneva, Geneva, Switzerland; University of Florida, United States of America

## Abstract

**Background:**

Ccr4-Not is a highly conserved multi-protein complex consisting in yeast of 9 subunits, including Not5 and the major yeast deadenylase Ccr4. It has been connected functionally in the nucleus to transcription by RNA polymerase II and in the cytoplasm to mRNA degradation. However, there has been no evidence so far that this complex is important for RNA degradation in the nucleus.

**Methodology/Principal Findings:**

In this work we point to a new role for the Ccr4-Not complex in nuclear RNA metabolism. We determine the importance of the Ccr4-Not complex for the levels of non-coding nuclear RNAs, such as mis-processed and polyadenylated snoRNAs, whose turnover depends upon the nuclear exosome and TRAMP. Consistently, mutation of both the Ccr4-Not complex and the nuclear exosome results in synthetic slow growth phenotypes. We demonstrate physical interactions between the Ccr4-Not complex and the exosome. First, Not5 co-purifies with the exosome. Second, several exosome subunits co-purify with the Ccr4-Not complex. Third, the Ccr4-Not complex is important for the integrity of large exosome-containing complexes. Finally, we reveal a connection between the Ccr4-Not complex and TRAMP through the association of the Mtr4 helicase with the Ccr4-Not complex and the importance of specific subunits of Ccr4-Not for the association of Mtr4 with the nuclear exosome subunit Rrp6.

**Conclusions/Significance:**

We propose a model in which the Ccr4-Not complex may provide a platform contributing to dynamic interactions between the nuclear exosome and its co-factor TRAMP. Our findings connect for the first time the different players involved in nuclear and cytoplasmic RNA degradation.

## Introduction

In eukaryotic cells RNAs are synthesized by 3 different RNA polymerases and they are extensively processed to reach their mature forms. In many cases the processing is accompanied by degradation of processed RNA fragments and degradation of aberrantly processed RNAs occurs through surveillance pathways. The exosome is a conserved multi-subunit 3′ to 5′ exoribonuclease complex that plays a central role in a large number of the pathways related to RNA degradation. It also plays a major role in RNA processing pathways, both in the cytoplasm and in the nucleus (for review see [1]). The targets for the exosome are multiple since the exosome contributes to the turnover of cytosolic mRNAs, the normal processing of nuclear rRNAs and snoRNAs, and it participates in surveillance mechanisms leading to degradation of aberrant forms of different RNAs in the nucleus. The eukaryotic exosome is made up of 9 core subunits, with 6 subunits carrying the fold of bacterial RNase PH, a phosphorolytic RNase, namely Rrp41, Rrp42, Rrp43, Rrp45, Rrp46 and Mtr3, and 3 putative RNA-binding proteins, Csl4, Rrp4 and Rrp40 [2]. It is constitutively associated with a processive hydrolytic exoribonuclease, Rrp44, which is the only active nuclease of the yeast core exosome [3]. In the nucleus, the core exosome is associated with 2 additional proteins, Rrp6 [4], another hydrolytic RNase, which provides hydrolytic activity to the nuclear exosome but might also function independently of the core exosome [5], and Rrp47, which cooperates with Rrp6 [6], [7]. In general, the activity of the exosome is regulated through association with co-factors such as Ski7 and the Ski2, 3, and 8 complex in the cytoplasm [8] or the TRAMP complex in the nucleus [9], [10], [11].

TRAMP is particularly important for surveillance pathways in the nucleus, where it is thought that it recognizes structural features of aberrant RNAs, leading to their selective polyadenylation, followed by recruitment and activation of the nuclear exosome (for review see [12]). TRAMP is composed of a poly(A) polymerase, either Trf4 or Trf5, a putative RNA binding protein, Air1 or Air2, and a 3′ to 5′ RNA helicase Mtr4 [9], [10], [11]. TRAMP also functions with the nuclear exosome to degrade cryptic non-protein coding transcripts generated by RNA polymerase II, rendering the transcripts highly unstable (CUTs) [13]. Recent evidence has demonstrated that such transcripts control transcription initiation of stable mRNAs by RNA polymerase II using different mechanisms [14], [15], [16], [17].

The Ccr4-Not complex is another conserved eukaryotic multisubunit complex whose activity has been associated both with RNA degradation and transcriptional regulation (for reviews see [18], [19]). Indeed, one of its subunits, Ccr4, is the major yeast deadenylase [20], which catalyzes the first step leading to the subsequent degradation of cytoplasmic mRNAs. This activity is supported by another Ccr4-Not subunit, Caf1. Besides Ccr4 and Caf1, the yeast Ccr4-Not complex has 7 other subunits, Caf40, Caf130 and 5 Not subunits, Not1-Not5. The only subunit that is essential for yeast viability is Not1, the scaffold of the complex [21]. Not4 is an E3 ligase whose only known substrate is the EGD/NAC complex [22], a chaperone associated with ribosomes that is in contact with nascent peptides [23], [24], [25]. Ubiquitination of this chaperone by Not4 was recently shown to be important for its association with the ribosome and with the proteasome [26]. The role of the other subunits has not been clearly defined. Several studies have demonstrated the importance of the Ccr4-Not complex for appropriate transcription initiation, in particular for the appropriate promoter distribution of the general transcription factor TFIID [27], and both genetic and physical interactions between the Ccr4-Not complex and TFIID have been described [28], [29]. Transcription functions of the Ccr4-Not complex are also consistent with the observation that a mutation in *NOT1* is suppressed by the deletion of *SPT3* [30], encoding a subunit of the SAGA co-activator complex [31], [32] and a protein that interacts with TBP. Though many studies have suggested roles of the Ccr4-Not complex in other cellular functions, such as resistance to ionizing radiation and DNA damage or replication stress [33], [34], mechanistic understanding of the connection between the Ccr4-Not complex and these other pathways is lacking. However, consistent with transcription and cytoplasmic RNA degradation functions, the Ccr4-Not complex, like the exosome, has been reported to have both cytoplasmic and nuclear localizations [20], [35]. Indeed, while association of certain subunits with promoters has been described [28], [36], the presence of others was revealed in cytoplasmic P bodies [37].

A recent investigation of the role of the 8 non-essential Ccr4-Not subunits in the expression of the yeast genome revealed that each subunit had a very specific function in the expression of the genome [38]. Surprisingly, an excessive presence of polyadenylated snRNAs and snoRNAs was highly and significantly identified in certain mutants. Such a phenotype is characteristic of nuclear exosome mutants that fail to degrade aberrant nuclear RNAs [39]. In this study we demonstrate genetic and biochemical interactions between the Ccr4-Not complex and the nuclear RNA degradation machinery provided by TRAMP and the nuclear exosome. Our findings shed a new light on the functional organization of the Ccr4-Not complex and connect the different players involved in cellular RNA degradation.

## Results

### Synthetic genetic interactions between the Ccr4-Not complex and the nuclear exosome

**Figure 1:**
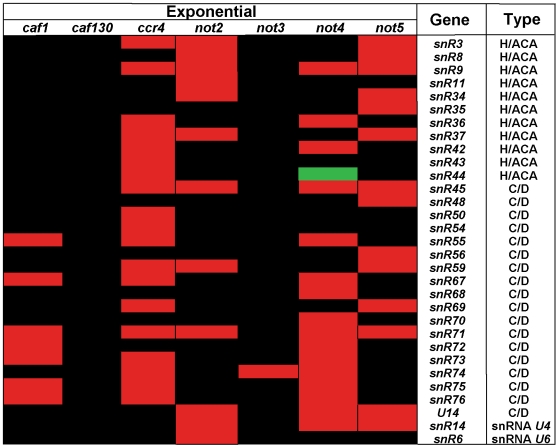
Polyadenylated non-coding snRNAs and snoRNAs are over-expressed in specific mutants of the Ccr4-Not complex. This figure lists all of non-coding snoRNAs and snRNAs identified in our previous micro-array experiments [38] as being differentially expressed in any mutant of the Ccr4-Not complex growing exponentially compared to the wild-type. Black, red and green shows genes that are not affected, over-expressed or under-expressed, respectively. In the last column is indicated which category the gene belongs to either a H/ACA or C/D snoRNA, or a snRNA.

Micro-array experiments designed to measure polyadenylated RNAs [38] revealed that *U4* and *U6* snRNAs, as well as many snoRNAs of all different types and origin (H/ACA or C/D boxes, intronic, mono-cistronic or polycistronic), were over-expressed in certain mutants of the Ccr4-Not complex, especially *ccr4Δ*, *not2Δ*, *not4Δ* and *not5Δ* (Fig. 1 and Table S1). These nuclear non-coding RNAs should in principle not be polyadenylated. However, transcripts for a wide variety of snoRNAs have been shown to accumulate as 3′-extended polyadenylated species in mutants of the nuclear exosome [39]. In this context it is interesting to note that we isolated Rrp6, an exonuclease specific of the nuclear exosome, as a two-hybrid partner of Not1 in a genome-wide screen performed several years ago (described in [27]). These observations from former studies led us to investigate a possible link between the Ccr4-Not complex and the nuclear exosome in this work.

**Figure 2:**
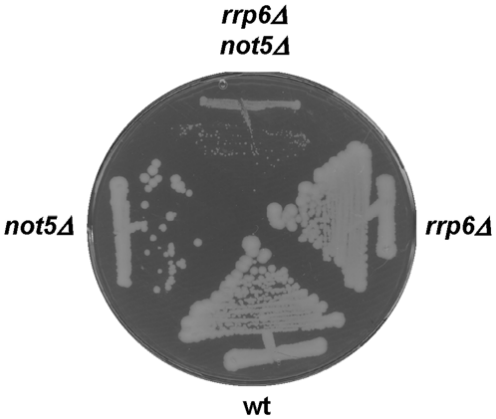
Synthetic growth phenotype when deletions of *RRP6* and *NOT5* are combined. The indicated strains were streaked on YPD plates and let to grow for several days at 30°C.

We created strains lacking Rrp6 and individual subunits of the Ccr4-Not complex. The double mutant strains displayed synthetic slow growth phenotypes when compared to the single mutants (Fig. S1). The most extreme phenotype was observed in the case of *not5Δ*, since the double mutant grew so poorly (Fig. 2) that it was very difficult to obtain a growth curve in liquid medium. To determine whether the synthetic growth phenotypes might correlate with synthetic defects in expression of nuclear snoRNAs, we studied the expression of one snoRNA identified in our micro-arrays, namely *U14*, in single or double mutant cells. Indeed, this snoRNA is processed from a polycistronic RNA [40] and is inappropriately processed and accumulates in larger polyadenylated forms in nuclear exosome mutants [41].

**Figure 3:**
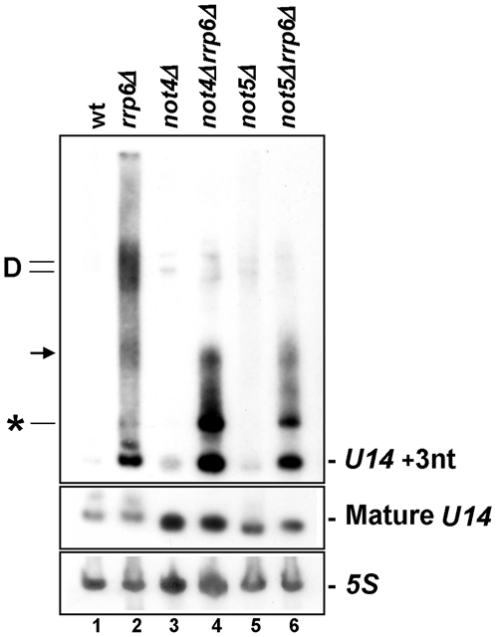
Accumulation of 3′-extended and polyadenylated *U14* in *rrp6Δ* cells is affected by the Ccr4-Not complex. Total cellular RNA isolated from the indicated strains was analyzed by northern blot first with a probe against 3′-extended *U14* (upper panel) and then with a probe for mature *U14* (middle panel) and a probe for *5S* rRNA (lower panel). The position of several extended *U14* forms and mature *U14* are indicated on each side of the blot (see the text for details). Hybridization against the *5S* mature rRNA is shown as a control for loading.

As expected from previous studies [6], [42], northern blot analysis with a probe specific to the sequence downstream of the 3′ end of mature *U14* revealed several 3′-extended heterogeneously polyadenylated forms of this snoRNA in cells lacking Rrp6 (Fig. 3, lane 2). In particular three stabilized *U14* 3′-extended-species were seen: the slowest migrating one indicated by D on the left of Fig. 3 may correspond to the dicistronic precursor thought to be stabilized in the absence of Rrp6 [43]. The second one indicated by an arrow is likely to be the 3′-extended *U14* precursor that does not get processed in the absence of Rrp6 [6], [42]. Finally, the third one indicated by a star has not been clearly identified, but its accumulation has been reported to be specific to deletions of Rrp6 or Rrp47 [6]. In the single mutants of the Ccr4-Not complex (Fig. 3, lanes 3 and 5), there appeared to be an accumulation of heterogenous *U14* that was faint in this experiment, but more visible in others (Fig. S2, panel A). Strikingly, when *NOT4* or *NOT5* were deleted in the *rrp6Δ* mutant (Fig. 3, lanes 4 and 6), the largest precursor D accumulated less than in the single *rrp6Δ* mutant, whereas the faster migrating 3′-extended *U14* precursor (indicated by a star) accumulated to a greater extent. It was difficult to assess the levels of precursor D in the *not4Δ* and *not5Δ* single mutants, though it seemed to accumulate somewhat (Fig. 3, lanes 3 and 5).

Hence, the profile of inappropriately processed and polyadenylated *U14* forms that accumulate in *rrp6Δ* cells is affected by concomitant mutations of the Ccr4-Not complex. We extended this analysis to several other snoRNAs, namely *U18*, *snR71* and *U3* and observed that similarly to the situation described for *U14*, the profile of extended snoRNAs that accumulate in *rrp6Δ* cells is affected by concomitant deletion of either Not4 or Not5 (Fig. S2 panel B).

### Integrity of the largest Rrp41-containing complexes depends upon the Ccr4-Not complex

**Figure 4:**
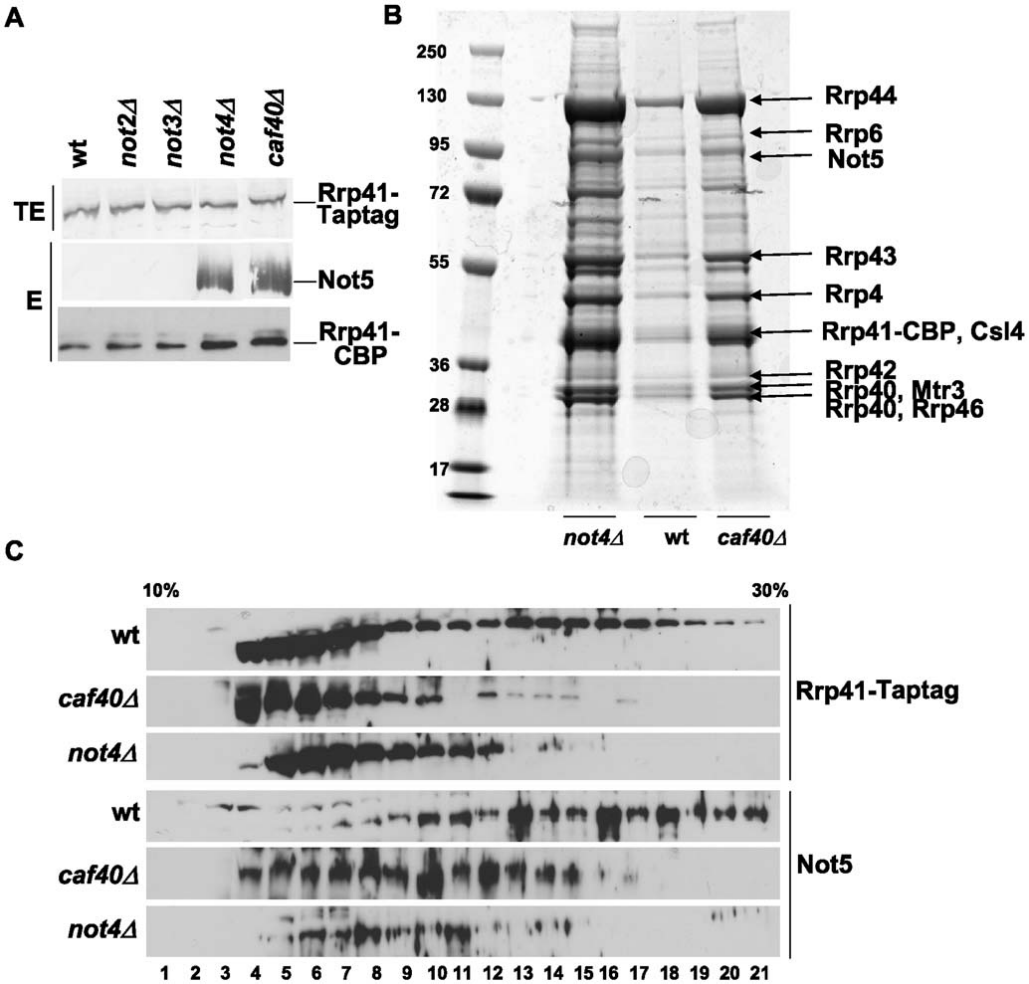
Large complexes containing Rrp41 and Not5 are disrupted in cells lacking Caf40 or Not4. A. Equal amounts of total protein extracts (TE) from the indicated strains expressing Tap-tagged Rrp41 were analyzed by western blot for the expression of Rrp41 with antibodies against CBP. Purified materials (E) were analyzed for the presence of Not5 and Rrp41 by western blotting with antibodies against Not5 and CBP as indicated. B and C. Wild-type, *caf40Δ* or *not4Δ* cells expressing Tap-tagged Rrp41 were grown for extract preparation. B. 2 g of extract were used to purify Rrp41 and the eluted proteins were loaded on SDS-PAGE. The gel was stained with coomassie. The nuclear exosome subunits and Not5 identified by mass spectrometry (Table S3) are indicated. Molecular weight markers are indicated on the left (in kDa). C. 5 mg of extracts were loaded on a 10–30% glycerol gradient. Proteins in the different fractions of the gradient were analyzed by western blotting for the presence of Tap-tagged Rrp41 with antibodies against CBP, and for Not5 with antibodies against Not5, as indicated.

This effect of the Ccr4-Not complex on accumulation of aberrant *U14* in the absence of Rrp6 led us to determine whether the Ccr4-Not complex might have an impact on the exosome. For this, we created wild-type and *ccr4-not* mutant strains expressing Tap-tagged Rrp41, a core exosome subunit. The expression of the tagged protein was similar in wild-type and mutant strains (Fig. 4A, upper panel) and the tagged protein could be affinity-purified from all strains (Fig. 4A, lower panel). Interestingly, a specific subunit of the Ccr4-Not complex, namely Not5, was recovered when Rrp41 was affinity purified from cells lacking Not4 or Caf40, but not from wild-type cells or from the other mutants (Fig. 4A, middle panel).

These observations led us to analyze the affinity-purified Rrp41 from wild-type, *not4Δ* or *caf40Δ* cells by separation on SDS-PAGE followed by mass spectrometry. We identified most of the exosome subunits, and the nuclear exosome subunit Rrp6, in all 3 purifications (Fig. 4B and Table S2), but the recovery of exosome seemed greater in the case of *not4Δ* and *caf40Δ* This was not due to increased expression of exosome subunits in mutants as verified by analyzing wild-type, *not4Δ* and *caf40Δ* strains expressing each of the exosome subunits as a Tap-tagged fusion protein (data not shown). The presence of Not5 in the purifications from the two mutants was confirmed by mass spectrometry (Table S2), and Not5 was not identified in the purification from the wild-type, supporting the analysis by western blot (Fig. 4A). Control affinity purifications performed with wild-type and *ccr4-not* mutant strains not expressing any Tap-tagged protein revealed the presence of a low level of proteins isolated non-specifically (Fig. S3), but none of them could be determined as exosome or Ccr4-Not subunits (data not shown).

To understand why Not5 co-purified with Rrp41 specifically in certain mutants of the Ccr4-Not complex, we determined the size distribution of Rrp41-containing complexes in wild-type and mutant cell extracts. This was investigated by glycerol gradient fractionation of total cell extracts followed by western blot analysis of the fractions with antibodies against the calmodulin binding peptide entity of the Tap-tag (CBP). In wild-type cells Rrp41 eluted with a very broad profile suggesting that it was present in complexes of very different sizes (Fig. 4C), including very large complexes (fractions 17–21 at the bottom of the gradient, in which both *25S* and *18S* rRNAs could be found (Fig. S4)). Not5 also eluted with a broad profile, consistent with previous studies using superose 6 gel filtration analyses of total protein extracts [21]. In particular, it was also present in fractions 17–21 (Fig. 4C). The deletion of Not4 and Caf40 lead to a loss of Not5 in the fractions corresponding to these largest complexes (see fractions 17–21 in Fig. 4C). This was not surprising for Not5 in *not4Δ*, since previous studies have already demonstrated a partial disruption of Ccr4-Not complexes in this deletion strain [21], [38], but it was new for Not5 in *caf40Δ*, since the importance of Caf40 for Ccr4-Not complex integrity has not yet been studied. Surprisingly however, these mutations of the Ccr4-Not complex also had a great impact on the elution profile of Rrp41, since the largest Rrp41-containing complexes were disrupted in *caf40Δ* and *not4Δ* (Fig. 4C, see fractions 13–21). Thus the more efficient purification of Rrp41 in *caf40Δ* and *not4Δ* in one hand and the co-purification of Not5 with Rrp41 in other hand are correlated with the release of Rrp41 from large soluble structures.

### RNA limits accessibility of the Not5-interacting exosome to purification

These results are compatible with the idea that the Not5-associated Rrp41 that was affinity purified in the mutants might have been released from the larger Rrp41 complexes in *not4Δ* and *caf40Δ*. However, it could also be that co-purification of Not5 with Rrp41 was detectable in the mutants simply because Rrp41 itself was more efficiently purified. Finally, the association of Not5 with Rrp41 could be an aberrant mutant phenotype resulting from the disruption of the Ccr4-Not complex.

**Figure 5:**
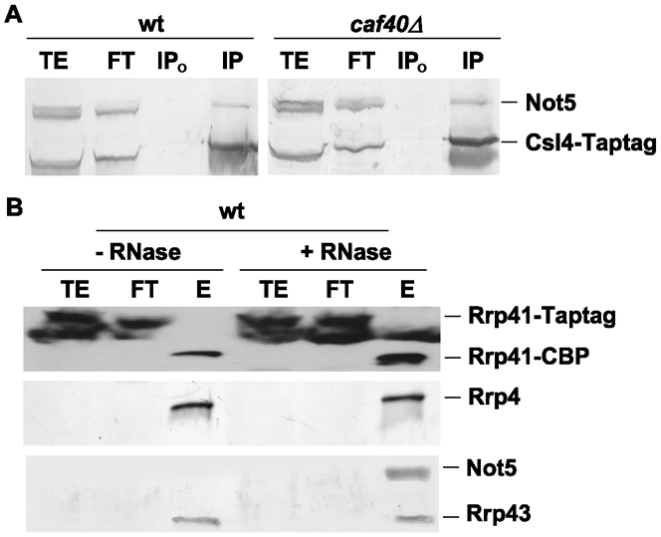
Not5 co-precipitates with Csl4 and Rrp41. A. Total protein extracts were prepared from wild-type or *caf40Δ* cells expressing Tap-tagged Csl4. 2 mg of total protein was incubated with (IP) or without (IP_0_) antibodies against CBP. 50 µg of total extract (TE), equivalent volume of unbound extract (FT) and the immunoprecipitate were loaded on SDS-PAGE followed by western blotting with antibodies against Not5, which revealed both Not5 and Tap-tagged Csl4 as indicated. B. 2 g of total protein extract from wild-type cells expressing Tap-tagged Rrp41 treated or not with RNAse as indicated were processed for tandem affinity purification of Rrp41. Equivalent amounts of total extract (TE), flow through (FT) or one fourth of the eluate precipitated by TCA, were loaded on SDS-PAGE and analyzed with antibodies against CBP to follow Rrp41, or against Rrp4, Rrp43 and Not5 as indicated.

To first exclude this latter possibility, and to additionally determine whether Not5 interacted with the entire exosome or only with Rrp41, we determined whether Not5 co-purified with any other exosome subunit in wild-type and mutant cells. Indeed, detectable levels of Not5 co-immunoprecipitated with Tap-tagged Csl4 from wild-type cell extracts and from extracts of cells lacking Caf40 (Fig. 5A). The purification of Tap-tagged Csl4 from total extracts of wild-type cells was generally more efficient than that of Rrp41 (data not shown) suggesting that the tag on Csl4 might be more accessible in the different exosome-containing complexes. In any event, these results indicate that Not5 is likely to interact with the exosome, and that this interaction occurs in wild-type cells. Indeed, Not5 could also be co-immunoprecipitated with several other Tap-tagged exosome subunits from wild-type cells (Fig. S5).

We next wanted to determine the nature of the large Rrp41-containing structures whose integrity was dependent upon the Ccr4-Not complex. In particular we wanted to determine whether they might contain RNA, since both the Ccr4-Not complex and the exosome are RNA-degrading machines. For this purpose, we prepared total protein extracts from wild-type cells expressing Tap-tagged Rrp41 that we treated or not with RNAse A prior to tandem affinity purification of Rrp41. In both cases the exosome was purified, as determined by western blot analysis with antibodies against CBP (to detect Rrp41), and against Rrp4 or Rrp43 (Fig. 5B). Not5 co-purified with Rrp41 from wild-type cell extracts treated with RNAse A but not from untreated wild-type cell extracts (Fig. 5B). Thus, in wild-type cell extracts RNA limits the accessibility of Not5-associated Rrp41. In good correlation with this finding, digestion of RNA in total cellular extracts prior to their separation by glycerol gradient centrifugation showed that the integrity of the largest Rrp41-containing complexes was indeed RNA-dependent (Fig. S6).

### The Ccr4-Not complex interacts with the exosome

**Figure 6:**
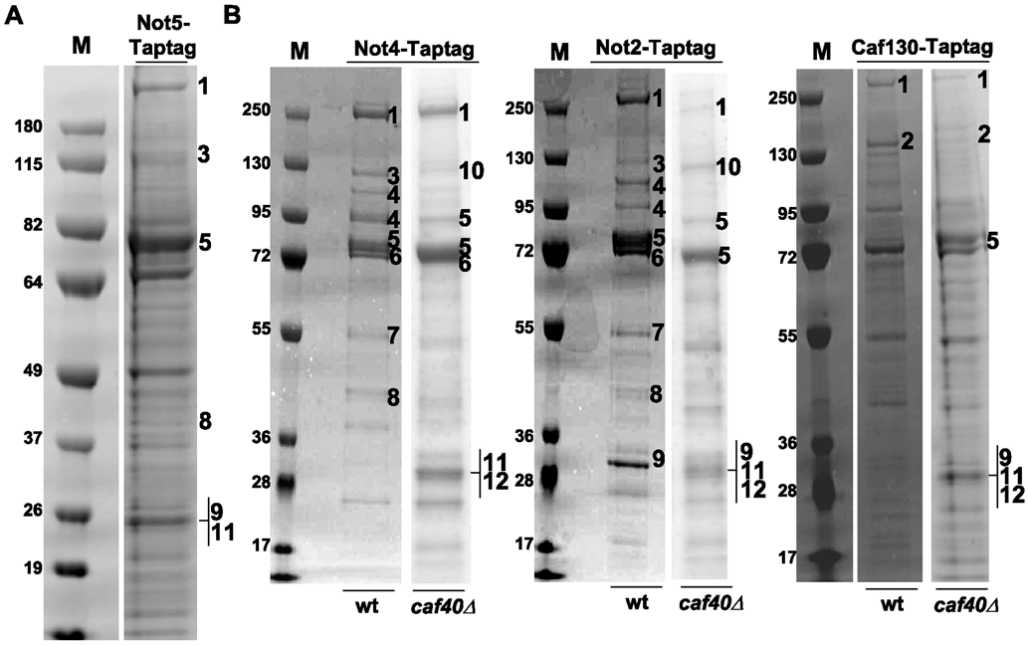
Subunits of the Ccr4-Not, exosome and TRAMP complexes co-purify. 2 g of total proteins extracted from (A) wild-type cells expressing Tap-tagged Not5 or (B) wild-type and *caf40Δ* cells expressing Tap-tagged Not4, Not2, or Caf130 as indicated, were subject to tandem affinity purification. After separation on SDS-PAGE, and coomassie staining, the purified proteins were identified by mass spectrometry analysis (see Table S3). The components of the Ccr4-Not, TRAMP or exosome complexes identified are indicated on the right of the gel lanes by numbers which refer to the following proteins: 1 is Not1, 2 is Caf130, 3 is Not3, 4 is Ccr4, 5 is Not5, 6 is Not4, 7 is Caf1, 8 is Caf40, 9 is Not2, 10 is Mtr4, 11 is Rrp41 and 12 is Rrp42. Molecular weight markers (M) are indicated on the left of the gels (in kDa).

The co-purification of Not5 with the exosome isolated via Rrp41 or Csl4 raised the question of whether the entire Ccr4-Not complex interacts with the exosome, or whether Not5 interacts with the exosome independently of the Ccr4-Not complex. Indeed to date it is still unclear whether Ccr4-Not subunits function outside the context of a complex or not. The improved recovery of the exosome by affinity purification of tagged Rrp41 when the Ccr4-Not complex was compromised supported the idea that the entire Ccr4-Not complex was involved, but its role could have been indirect. To address this issue, we first purified Tap-tagged Not5 itself and identified Not1, Not2, Not3, and Caf40, as well as Rrp41, as co-purifying proteins (Fig. 6A and Table S3). These results confirmed an interaction between Not5 and Rrp41, but did not clearly determine whether the Ccr4-Not subunits co-purifying with Not5 were within the same complex as Rrp41. We next tandem-affinity purified the Ccr4-Not complex through different subunits from wild-type cells or from the *caf40Δ* mutant in which accessibility of the Not5-exosome complexes was improved (see above). Depending upon which subunit of the Ccr4-Not complex was tagged, the success in purifying intact Ccr4-Not complexes from wild-type cells was variable. For instance, purification of Tap-tagged Not4 led to isolation of complexes lacking Not2 and Caf130, purification of Tap-tagged Not2 led to isolation of complexes lacking Caf130, whereas purification of Tap-tagged Caf130 was inefficient, and only led to co-purification of Not1 (Fig. 6B). The deletion of Caf40 had a major impact in all cases. For Not2 or Not4, the co-purification of several subunits of the Ccr4-Not complex, namely Not3, Ccr4 and Caf1, was less efficient, suggesting that the core Ccr4-Not complex might be less stable in the absence of Caf40. In contrast, for Caf130 the co-purification of Not2 and Not5 was greater, suggesting that the tag on Caf130 within Ccr4-Not complexes might have become more accessible because of the Caf40 deletion. Most importantly, for the *caf40Δ* strains, Rrp41 and Rrp42 co-purified with the Tap-tagged Ccr4-Not subunits (Fig. 6B and Table S3). Thus, the isolation of exosome subunits by purifying different Ccr4-Not subunits besides Not5 suggests that these exosome subunits most likely interact with the integral Ccr4-Not complex.

### Mtr4 of TRAMP interacts with the Ccr4-Not complex

**Figure 7:**
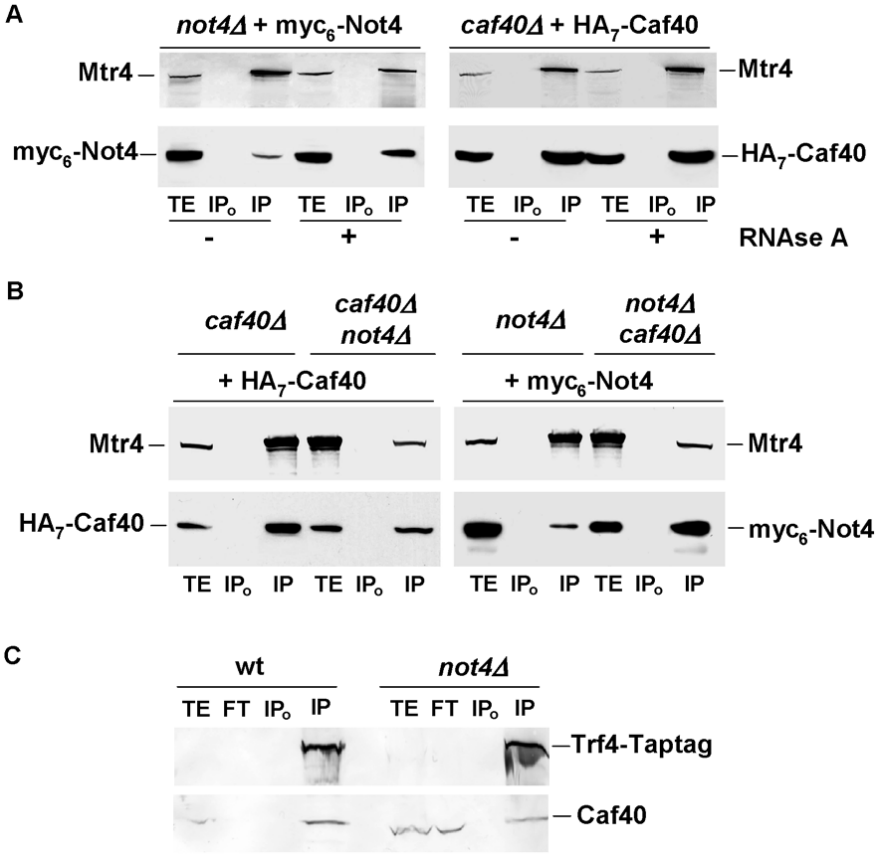
Mtr4 co-immunoprecipitates with subunits of the Ccr4-Not complex independently of RNA. A. Equivalent amounts of total protein extract (5 mg) treated or not (+ or −) with RNAse A as indicated, prepared from *not4Δ* cells expressing complementing myc_6_-Not4 from an episome or *caf40Δ* cells expressing complementing HA_7_-Caf40 from an episome, were immunoprecipitated with antibodies against Mtr4. Immunoprecipiates were analyzed by western blotting for the presence of Mtr4 and tagged Not4 or Caf40 as indicated. The efficient digestion of RNA in total extracts (TE) was verified on an agarose gel stained with ethidium bromide (see Fig. S7). B. The same experiment as in A was performed except *caf40Δ not4Δ* cells expressing complementing myc_6_-Not4 from an episome or *not4Δ caf40Δ* cells expressing complementing HA_7_-Caf40 were analyzed. C. 2 mg of total protein extracts from wild-type or *not4Δ* cells expressing Tap-tagged Trf4 were incubated with (IP) or without (IP_0_) antibodies against CBP. 50 µg of total extract (TE), equivalent volume of unbound extract (FT) and the immunoprecipitate were analyzed by western blotting with antibodies against Caf40, which revealed both Caf40 and Tap-tagged Trf4 as indicated.

The purification of Not2 and Not4 from *caf40Δ* cell extracts also led to the co-purification of Mtr4, an RNA helicase of the TRAMP complex, which serves as a co-factor for the nuclear exosome (Fig. 6B and Table S3). Mtr4 also co-purified with Tap-tagged Caf40 from wild-type cell extracts (data not shown). To confirm this interaction between Mtr4 and the Ccr4-Not complex, Mtr4 was immunoprecipitated from extracts prepared from *not4Δ* cells expressing myc-tagged Not4 from an episome or from *caf40Δ* cells expressing HA-tagged Caf40 from an episome. Both Caf40 and Not4 co-immunoprecipitated with Mtr4, and these interactions were RNA-independent (Fig. 7A and Fig. S7). We also observed the co-immunoprecipitation of other subunits of the Ccr4-Not complex (Ccr4, Not3, Not5 and Caf1) with Mtr4, using Tap-tagged Mtr4, and polyclonal antibodies against the Ccr4-Not complex subunits (data not shown). RNA degradation had no effect on the co-precipitation of Caf40 with Mtr4, whilst the co-precipitation of Not4 with Mtr4 was improved. Since Mtr4 was the bait protein each time, these results suggested that complexes containing Mtr4 and Caf40 might not entirely overlap those containing Mtr4 and Not4. We hence tested the association of Mtr4 with Not4 in *caf40Δ*, and the association of Mtr4 with Caf40 in *not4Δ*. The deletion of Not4 led to a reduced co-precipitation of Mtr4 with Caf40. In contrast, the co-precipitation of Mtr4 with Not4 was improved in the absence of Caf40 (Fig. 7B) and the same was observed for the co-precipitation of Mtr4 with the other subunits of the Ccr4-Not complex mentioned above (not shown). Hence Not4 contributes to the interaction between Caf40 and Mtr4, but Caf40 seems to be in competition with the interaction between Mtr4 and other subunits of the Ccr4-Not complex. Interestingly, Caf40 co-immunoprecipitated with another subunit of the TRAMP complex, the Trf4 polymerase, and this co-immunoprecipitation was also decreased in cells lacking Not4 (Fig. 7C). In addition, Not5 co-immunoprecipitated not only with Tap-tagged Mtr4 (as mentioned above) and with Tap-tagged Air1 (data not shown) but also with Tap-tagged Air2 (Fig. S5). These results suggest that the Ccr4-Not complex interacts with TRAMP and that probably in this context Not4 contributes to the association of Caf40 with TRAMP.

### Association of Mtr4 with Rrp6 depends upon Caf40

**Figure 8:**
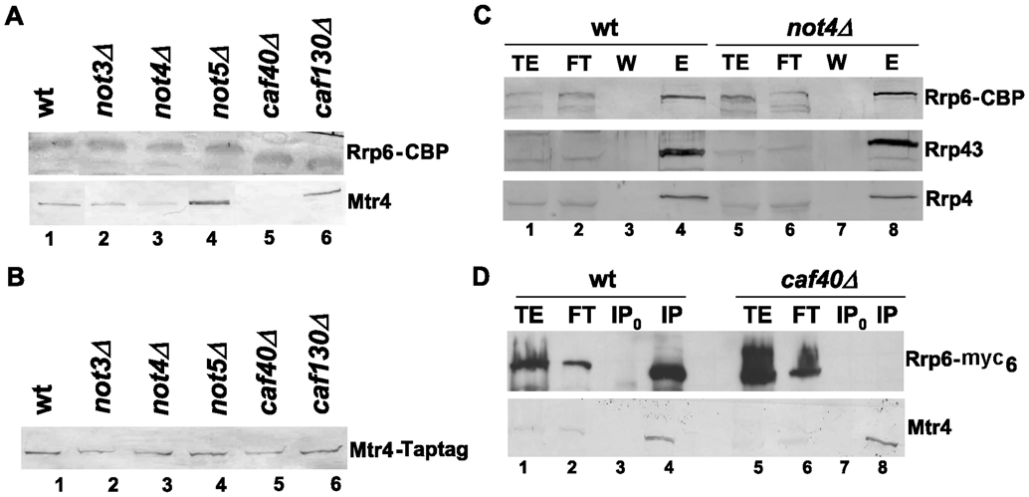
Co-purification of Mtr4 with Rrp6 depends upon the Ccr4-Not complex. A. Total protein extracts were prepared from wild-type and mutant strains expressing Tap-tagged Rrp6. The same amount of each extract (60 mg) was incubated with IgG beads. After washing, bound proteins were eluted by TEV cleavage and analysed by western blot with anti-CBP antibodies (upper panel) or with polyclonal anti-Mtr4 antibodies (bottom panel). B. Equivalent amounts of total protein extracts from the indicated strains expressing Tap-tagged Mtr4 were analyzed by western blot for the presence of Tap-tagged Mtr4 with antibodies against Mtr4. C. 2 g of total proteins extracted from wild-type or *not4Δ* cells expressing Tap-tagged Rrp6 were incubated with IgG beads, and bound proteins were eluted with TEV protease. Co-purified proteins were analyzed by western blotting for the presence of Rrp43, Rrp4, or Rrp6 with antibodies against CBP. D. Total protein extracts were prepared from wild-type and *caf40Δ* cells expressing myc-tagged Rrp6. The same amount of extract was incubated with antibodies against Mtr4 and immunoprecipitates were analyzed by western blotting with antibodies against myc. The same blot was then analyzed with antibodies against Mtr4.

The results presented so far show that the Ccr4-Not complex can interact with the nuclear exosome on one hand and with Mtr4 on the other hand. This led us to question whether it might play a role in the interaction of the nuclear exosome with its co-factor. To address this question, we created wild-type and *ccr4-not* mutant strains expressing Tap-tagged Rrp6 and immunopurified Rrp6 using a single affinity step. Equal levels of Rrp6 were purified from all strains (Fig. 8A, upper panel), and Mtr4 generally co-purified with Rrp6, but to reduced levels from *not4Δ* compared to wild-type cells and not at all from *caf40Δ* (Fig. 8A, lower panel). However, expression of Mtr4 was similar in wild-type and *ccr4-not* mutant strains (Fig. 8B), and the co-purification of Rrp6 with the exosome was similar in wild-type and mutant strains (shown for *not4Δ* in Fig. 8C). Thus, it seems that the association of Mtr4 with Rrp6 might be compromised in *caf40Δ*. To confirm this finding we created wild-type and *caf40Δ* strains expressing myc-tagged Rrp6, and immunoprecipitated Mtr4 to determine the presence of Rrp6 in the immunoprecipitate. Though equal levels of Mtr4 were immunoprecipitated from both strains, no co-precipitation of Rrp6 with Mtr4 was detected in *caf40Δ* (Fig. 8D). Thus, the interaction between Mtr4 and Rrp6 is indeed compromised in *caf40Δ*.

## Discussion

### The Ccr4-Not complex is physically and functionally connected to TRAMP and the nuclear exosome

In this work, we have made the exciting finding that the Ccr4-Not complex, whose role in cytoplasmic RNA degradation has been clearly demonstrated [20], is also connected to the nuclear RNA degradation machinery. Starting from our finding in a previous study of a very significant accumulation of polyadenylated snoRNAs in several mutants of the Ccr4-Not complex [38] we observed synthetic growth phenotypes when mutants of the Ccr4-Not complex were combined with the deletion of the nuclear exonuclease Rrp6, whose role in processing and degradation of aberrantly processed snoRNAs has been clearly established. These genetic interactions are consistent with our identification of a remarkable alteration in the profile of aberrantly processed and polyadenylated *U14* snoRNA in *rrp6Δ*when the Ccr4-Not complex was mutated. We could determine that these genetic and functional interactions correlate with physical interactions between the Ccr4-Not complex and the nuclear RNA degradation machinery provided by TRAMP and the nuclear exosome. Indeed, we demonstrated that Not5 co-purified with the exosome through Rrp41 or Csl4, that the purification of Ccr4-Not complexes through different subunits led to the co-purification of the Rrp41 and Rrp42 subunits of the exosome, and that the association of the exosome subunit Rrp41 in very large soluble structures was dependent upon subunits of the Ccr4-Not complex. We also provided evidence that the Ccr4-Not and exosome complexes are likely to be associated in RNA-containing structures. Finally, we showed that the Ccr4-Not complex was physically associated with the Mtr4 RNA helicase, a subunit of the TRAMP complex and co-factor of the nuclear exosome.

Though we did not demonstrate that Mtr4 and Rrp41 were a part of the same Ccr4-Not containing complex, it is interesting to note that Mtr4 and exosome subunits were purified together with the Ccr4-Not complex in the same affinity purifications. At present we cannot be sure that these interactions occur within a single soluble structure, but these results are nevertheless compatible with a model in which the Ccr4-Not complex physically connects the nuclear exosome and its co-factor Mtr4 of TRAMP. Because the exosome and Ccr4-Not interactions occur in RNA-containing structures, it is very tempting to speculate that the Ccr4-Not complex may contribute to the dynamic assembly of the nuclear RNA degradation machinery with its target RNAs.

It may seem surprising that such interactions have not been described so far, despite extensive work and purification of both TRAMP and exosome complexes in many different studies, including [3], [4], [6], [9], [10], [44], [45], [46], [47]. However, we identified these interactions first in situations where the integrity of the Ccr4-Not complex was perturbed, situations in which the exosome and TRAMP have not been purified previously.

### Not5 and Rrp41 connect the exosome and the Ccr4-Not complex

As mentioned above, our study revealed a series of physical interactions between Ccr4-Not, exosome or TRAMP subunits. However curiously, when purifying the Ccr4-Not complex in different ways, we only co-purified at most a couple of the exosome subunits and only Mtr4 of TRAMP, and similarly when purifying the exosome, we only co-purified Not5 of the Ccr4-Not complex. This raises the question of whether the partial entities that we isolated exist as such *in vivo*. However, we believe that this is highly unlikely and rather imagine that *in vivo* the Ccr4-Not complex interacts with the integral exosome and maybe also the integral TRAMP complex. Nevertheless, we cannot exclude that the Ccr4-Not complex might serve as some form of a chaperone for TRAMP and/or the exosome *in vivo*, and that this explains why it can be isolated interacting only with certain subunits of these complexes. Alternatively, it could be that the interactions between these entities are highly dynamic and only stabilized in the largest structures where however the accessibility to purification of the different components is reduced. Hence, partial disruption of these structures required to access the different components may also disrupt or destabilize certain entities such that only the directly interacting partners are captured during purification. If this is the case, according to the different analyses that we performed, Not5 and Rrp41 would be the subunits within each complex that directly interact. Rrp42 was isolated in certain cases with Rrp41 and the Ccr4-Not complex and may be closely connected to Rrp41 within the exosome. This is certainly supported by the three dimensional interaction map derived by isolating exosome complexes directly from cells [48]. It will be interesting to see whether an interaction between Rrp41 and Not5 can indeed be recapitulated *in vitro*.

In the case of the interactions between the Ccr4-Not complex and TRAMP, Mtr4, but none of the other subunits of TRAMP were identified by mass spectrometry in any of our purifications, suggesting that it is the most direct partner of the Ccr4-Not complex. Concerning which subunit of the Ccr4-Not complex may be the privileged partner of Mtr4, this is still an open question.

It is clear that none of our experiments have actually determined that the interactions between the Ccr4-Not complex and either the exosome or TRAMP are directly mediated by interactions between subunits of these different complexes. However we have no evidence for the presence of additional proteins in our purifications that might serve as adaptors and RNA is not required for the interactions. *In vitro* experiments will nevertheless be required to determine this more precisely.

### A role for the Ccr4-Not complex in functional connection the nuclear exosome and TRAMP

This study has demonstrated an RNA-independent interaction between Not5 and the exosome, and between Mtr4 and the Ccr4-Not complex. It has also revealed the importance of certain Ccr4-Not complex subunits for co-purification of the nuclear exosome with its co-factor Mtr4. In parallel, non-coding nuclear RNAs whose turnover and/or processing needs the nuclear degradation machinery are also affected by the Ccr4-Not complex. An exciting implication of these findings is that the Ccr4-Not complex might be important to connect Mtr4 with the nuclear exosome for processing and/or degradation of their target RNAs.

**Figure 9:**
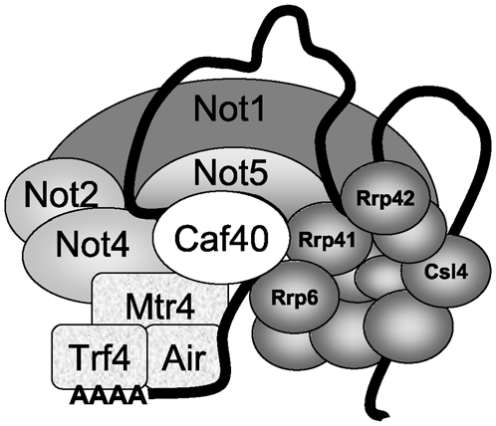
Model for the connections between the Ccr4-Not, TRAMP and exosome complexes. A large(s) structure(s) containing Ccr4-Not, TRAMP, the nuclear exosome and RNA, that may function to coordinate activity of TRAMP and the nuclear exosome, is suggested by our work. Not1, the scaffold of the Ccr4-Not complex is associated with Not5, Not2, Not4 and Caf40 (shown), as well as Caf1, Ccr4, Caf130, and Not3 (not shown). Mtr4 is a subunit of TRAMP that co-immunoprecipitates with Caf40 and Not4, and is co-purified with Tap-tagged Caf40 together with the Ccr4-Not complex. Its association with Not4 (and other subunits of the Ccr4-Not complex) increases in the absence of Caf40. In contrast, Caf40 is required for the interaction between Mtr4 and Rrp6, suggesting that maybe Caf40 contributes to release Mtr4 (and TRAMP) from the Ccr4-Not complex to the nuclear exosome. Rrp41 co-purifies with Tap-tagged Not5, and Rrp41, Rrp42 and Mtr4 co-purify with Not2-Taptag or Not4-Taptag when Caf40 is deleted, probably because the tagged proteins in association with the exosome and TRAMP become accessible to purification in this mutant. Finally, the nuclear exosome becomes more accessible to purification via Rrp41-Taptag when the integrity of the Ccr4-Not complex is compromised by deleting Caf40 or Not4, or when RNA is destroyed. Not5 co-purifies with Tap-tagged Csl4, and with Tap-tagged Rrp41 when it is more accessible to purification by RNA degradation or deletion of Caf40 or Not4.

Two subunits of the Ccr4-Not complex come out as particularly interesting in this functional association of the Ccr4-Not complex with the nuclear RNA degradation machinery. Not5, on one hand, co-purifies with the exosome, and its deletion is nearly lethal in cells lacking Rrp6. It might play a key role to bring the exosome to the Ccr4-Not complex. Caf40, on the other hand, is important for the integrity of the largest exosome-containing soluble structures and for the interaction between Rrp6 and Mtr4, but it competes for the interaction between Mtr4 and the other subunits of the Ccr4-Not complex. An exciting model (Fig. 9) is that the Caf40-Mtr4 interaction might be key to establish interactions between RNA-bound TRAMP, the Ccr4-Not complex and the nuclear exosome, but then also to transfer Mtr4 (and TRAMP) to the nuclear exosome, and to release it from the Ccr4-Not complex. In other words it might ensure dynamic interactions between all of these components. Our observation that the *not5Δ rrp6Δ* double mutant on one hand, and *not5Δ caf40Δ* double mutant on the other hand (unpublished data) have severe growth defects is compatible with the idea that Not5 and Caf40 might participate at different steps in the functional connection of TRAMP and the nuclear exosome.

Obviously, at present the mechanism by which the Ccr4-Not complex may be contributing to nuclear RNA expression with the nuclear exosome remains to be determined. Whether it may serve strictly as a platform allowing the appropriate assembly of the different partners, namely precursor RNA, nuclear exosome and TRAMP, or plays roles after processing, or even plays more direct roles for instance through the Ccr4 deadenylase, or by connecting nuclear events to the cytoplasm, really needs to be addressed. It will be interesting in a next step to confirm the roles attributed to Not5, Rrp41, Caf40 and Mtr4 in our model, and determine whether disruption of the proposed interactions will have an impact on nuclear exosome function. Finally, because it is becoming increasingly clear that unstable transcripts degraded by the nuclear exosome machinery contribute to regulate transcription, it will also be interesting to determine how much of the transcriptional phenotypes attributed to the Ccr4-Not complex might be indirectly due to inappropriate nuclear exosome function.

## Materials and Methods

### Strains, plasmids and media

The strains used in this study are listed in Table 1. All media were standard, either YPD for glucose rich medium, or synthetic complete media when the presence of a plasmid was selected for. Single-step deletions or tagging were performed by PCR as described by Longtine [49]. All of the strains were checked with a PCR reaction performed on genomic DNA extracts using a primer localized in the marker gene and a primer localized at the 5′ non-coding sequence of the target gene. All primers used are available upon request. Many new strains were created by crosses followed by sporulation and tetrad dissection. Plasmids expressing N-terminally tagged Not4 from the *NOT4* promoter (pMAC684) and Caf40 from the *SPT3* promoter (pMAC728) were made by PCR and homologous recombination, using the Drag and Drop system [50], and verified by sequencing.

**Table 1:** Strain list.

MY1	*MATa gcn4Δ ura3-52 trp1Δ1 leu2::PET56 gal2*	[52]
MY2182	Isogenic to MY1 except *not2::KanMX4 MATα*	[38]
MY4184	Isogenic to MY1 except *not3::KanMX4 MATα*	This work
MY3595	Isogenic to MY1 except *not4::KanMX4 MATα*	[51]
MY1719	Isogenic to MY1 except *not5::LEU2 MATα*	[38]
MY5129	Isogenic to MY1 except *caf1::TRP1 MATα*	[38]
MY4747	Isogenic to MY1 except *ccr4::TRP1 MATα*	[38]
MY5097	Isogenic to MY1 except *caf40::TRP1 MATα*	[38]
MY5091	Isogenic to MY1 except *caf130::TRP1 MATα*	[38]
MY3893	Isogenic to MY1 except *rrp6::TRP1*	This work
MY4849	Isogenic to MY1 except *rrp6::TRP1 not2::KanMX4*	This work
MY4820	Isogenic to MY1 except *rrp6::TRP1 not3::KanMX4*	This work
MY3980	Isogenic to MY1 except *rrp6::TRP1 not4::KanMX4*	This work
MY4063	Isogenic to MY1 except *rrp6::TRP1 not5::LEU2*	This work
MY5644	Isogenic to MY1 except *rrp6::TRP1 caf40::TRP1*	This work
MY5294	Isogenic to MY1 except *rrp6::TRP1 caf130::TRP1 MATα*	This work
MY4773	Isogenic to MY1 except *rrp6::TRP1 ccr4::TRP1*	This work
BY4741	*MATα leu2Δ20 ura3Δ met15Δ his3Δ1*	[53]
MY5906	Isogenic to BY4741 except *not2::NATMX4*	[38]
MY5241	Isogenic to BY4741 except *not3::HIS3MX4*	[38]
MY4910	Isogenic to BY4741 except *not4::HIS3MX4*	[38]
MY5673	Isogenic to BY4741 except *not5::NATMX4*	[38]
MY5267	Isogenic to BY4741 except *caf1::HIS3MX4*	[38]
MY5906	Isogenic to BY4741 except *caf40::HIS3MX4*	This work
MY5242	Isogenic to BY4741 except *caf130::HIS3MX4*	[38]
W303-1a	*MATa ade2-1 can1-100 his3-11,15 leu2-3,112 trp1-1 ura3-1*	[54]
MY5109	Isogenic to W303-1a, except *rrp6::RRP6-Tap-tag-TRP1*	[55]
MY5752	*rrp6::RRP6-Tap-tag-TRP1 not2::KanMX4*	From MY5109 x MY2182
MY5588	*rrp6::RRP6-Tap-tag-TRP1 not3::KanMX4*	From MY5109 x MY4184
MY5731	*rrp6::RRP6-Tap-tag-TRP1 not4::KanMX4*	From MY5109 x MY3595
MY5597	*rrp6::RRP6-Tap-tag-TRP1 not5::LEU2*	From MY5109 x MY1719
MY5628	*rrp6::RRP6-Tap-tag-TRP1 caf1::TRP1*	From MY5109 x MY5129
MY5636	*rrp6::RRP6-Tap-tag-TRP1 caf40::TRP1*	From MY5109 x MY5097
MY5647	*rrp6::RRP6-Tap-tag-TRP1 caf130::TRP1*	From MY5109 x MY5091
MY4858	Isogenic to BY4741 except *caf40::CAF40-Tap-tag-URA3*	[51]
MY5026	Isogenic to BY4741 except *not2::NOT2-Tap-tag-KanMX4 MATa*	This work
MY4857	Isogenic to BY4741 except *not4::NOT4-Tap-tag-URA3 MATa*	[51]
MY5711	Isogenic to MY5026 except *caf40::HIS3MX4*	MY5026 x MY5906
MY6017	Isogenic to MY4857 except *caf40::HIS3MX4*	MY4857 x MY5906
MY5218	Isogenic to BY4741 except *caf130::CAF130-Tap-tag-HIS3MX4*	This work
MY6016	Isogenic to MY 5218 except *caf40::HIS3MX4*	This work
MY5320	Isogenic to BY4741 except *not5::NOT5-Tap-tag-KanMX4*	This work
MY6426	*MATa ade2 arg4 leu2-3,112 trp1-289 ura3-52 rrp41::RRP41-Tap-tag-URA3*	Euroscarf
MY6561	*MATa rrp41::RRP41-Tap-tag-URA3 not4::KanMX4*	From MY6426 x MY3595
MY6630	*MATa rrp41::RRP41-Tap-tag-URA3 caf40::TRP1*	From MY6426 x MY5097
MY6732	*MATa rrp41::RRP41-Tap-tag-URA3 not2::NatMX4*	This work
MY6508	*MATa rrp41::RRP41-Tap-tag-URA3 not3::HIS3MX4*	This work
MY5562	*MATa ade2 arg4 leu2-3,112 trp1-289 ura3-52 mtr4::MTR4-Tap-tag-URA3*	Euroscarf
MY5873	*MATa mtr4::MTR4-Tap-tag-URA3 not3::KanMX4*	From MY5562 x MY4184
MY5741	*MATa mtr4::MTR4-Tap-tag-URA3 not4::KanMX4*	From MY5562 x MY3595
MY5742	*MATa mtr4::MTR4-Tap-tag-URA3 not5::NATMX4*	From MY5562 x MY5673
MY5871	*MATα mtr4::MTR4-Tap-tag-URA3 caf40::TRP1*	From MY5562 x MY5097
MY5937	*MATα mtr4::MTR4-Tap-tag-URA3 caf130::TRP1*	From MY5562 x MY5091
MY5926	*MATa mtr4::MTR4-Tap-tag-URA3 ccr4::TRP1*	From MY5562 x MY4747
MY6992	Isogenic to BY4741 except *csl4::CSL4-Tap-tag-URA3*	Euroscarf
MY7025	*MATa csl4::CSL4-Tap-tag-URA3 caf40::TRP1*	From MY6992 x MY5097
MY7029	*MATa csl4::CSL4-Tap-tag-URA3 not4::KanMX4*	From MY6992 x MY3596
MY7088	Isogenic to MY5097 except *MATa not4::KanMX4*	This work

### Northern blot analyses

For analysis of snoRNA steady-state levels, total cellular RNA was separated on an 8% polyacrylamide denaturing gel and electro-transferred to a nylon membrane in 0.5X TBE buffer. After cross-linking of the RNAs to the membrane by UV radiation, the membrane was hybridized with radio-labelled probes specific for the different mature or 3′extended snoRNAs or rRNA transcripts (sequences available upon request).

### Tandem affinity purification

Large-scale tandem affinity purifications from wild-type or mutant cells were performed as previously described with 2 g of total protein extract [51] except that the NaCl concentration was 150 mM. Small-scale purifications with Tap-tagged proteins were essentially done the same way except that only 2 l of cells were grown, and 60 mg of protein extracts processed with the volumes scaled down. Elution with TEV protease was performed with 1 ml, of which 200 µl were directly analyzed after TCA precipitation on SDS-PAGE followed by coomassie staining and 50 µl were analyzed for western blotting.

### Mass spectrometry

For mass spectrometry analysis the sample preparation, gel separation and gel staining were performed according to the SWISS-2D-PAGE protocols. Briefly, the samples were separated on 4–12% SDS-PAGE gradient gels and stained using coomassie in keratin free conditions. The visualized bands were excised from the gel and treated for mass spectrometry fingerprint analysis as following: they were washed 15 min at RT with acetonitril 30%, incubated 35 min at 56°C in 1,4,-dithioerythritol 10 mM, followed by 30 min incubation in iodoacetamid 55 mM at room temperature in the dark. After one wash for 10 min with ammonium biocarbonate 50 mM pH 8.0 and two other washes of 10 min with acetonitril 30%, samples were then completely dried down in a vacuum centrifuge for 1 h. After sample rehydration in 20 µl of freshly prepared digestion buffer containing 6.25 ng/µl trypsin for 45 min on ice, digestion was allowed to proceed over night at 37°C. To extract the peptides from the gel digestion supernatant was recovered and pooled with two successive washes of 20 min using 40 µl trifluoroacetic acid (TFA) 1% and one wash using TFA 0.1%. The collected supernatants were lyophilized to remove salts and washed again with 35 µl TFA 0.1% before sample concentration to 2–5 µl. 1 µl was loaded on a MALDI plate and after matrix addition, sample acquisition was performed using the MALDI-Tof MS (VOYAGEUR CONTROL PANEL program) and peptides were submitted to fingerprinting analysis. The obtained spectra were finally analyzed using the DATA EXPLORER program and proteins were identified using the MASCOT SEARCH website. When not provided as supplementary data, all identified peptides and scores are available upon request.

### Co-immunoprecipitation experiments

100 ml of cells expressing myc_6_-tagged Not4 or HA_7_-tagged Caf40 from plasmids were collected at an OD_600_ of 1.0 and broken with glass beads in 0.5 ml of buffer A (150 mM potassium acetate pH 7.5, 20 mM TrisHCl pH 8.0, 5 mM MgCl_2_, 0.1% Triton X-100, 1 mM DTT, 0.5 mM PMSF, Roche Anti Protease tablets) and spun at 16'000 g for 30 min at 4°C. Lysates were brought to equivalent protein concentration and 30 µl of lysates were boiled with 30 µl of 2 times concentrated SDS-sample buffer and used as total extracts (TE). 0.4 ml of the lysates containing 5 mg of total protein were treated or not with RNAse A (final concentration 1 µg/ml) for 5 min at room temperature and then were incubated with 20 µl of Protein-G-magnetic beads overnight at 4°C with (IP) or without (IP_0_) antibodies against Mtr4. Beads were washed three times with 1 ml buffer A and boiled with 40 µl of 2 times concentrated SDS-sample buffer. To analyze the proteins co-immunoprecipitated with Mtr4, 20 µl of total extracts (TE) and 20 µl of immunoprecipitates (IP) were loaded on an SDS-PAGE gel followed by western blotting with antibodies against the tag. To analyze the efficiency of RNAse A treatment, RNA was extracted from total extracts and loaded on a 1% agarose gel stained by ethidium bromide.

For cells expressing Tap-tagged proteins, extracts were prepared in 40 mM Hepes pH 7.5, 1 mM EDTA, 20% glycerol, 150 mM potassium acetate, 100 mM potassium chloride and protease inhibitors. 2 mg of extracts in 210 µl buffer were incubated overnight at 4°C with 40 µl of magnetic beads coupled to protein G equilibrated in the same buffer with or without 1 µl of antibody against CBP. After washing, the proteins were eluted from the beads in 20 µl of 2 times concentrated SDS-sample buffer.

### Glycerol gradient analysis

Total protein extracts (5 mg) were submitted to size separation on a 10 to 30% glycerol gradient in A200 buffer (200 mM potassium acetate, 20 mM Tris/HCl pH 8.0, 5 mM Mg acetate, 1 mM DTT). The gradients were spun at 4°C for 10 h at 20000 rpm in Beckmann Sw41 Ti rotor. 500 µl fractions were collected and proteins were extracted from 250 µl by 30 min precipitation in 10% TCA followed by resuspension in 30 µl of SDS loading buffer and 10 µl were analyzed by SDS-PAGE and western blotting. RNA was extracted from the other 250 µl by phenol chloroform extraction and ethanol precipitation and analyzed by migration on a 1% agarose gel.

## Supporting Information

Figure S1 Synthetic growth phenotypes when deletions of RRP6 and the Ccr4-Not complex are combined. The indicated strains were grown at 30°C exponentially in high glucose for 24 hours, then diluted to an OD600 of 0.2 and followed for growth during the next 11 hours by measuring the OD600.
(0.23 MB TIF)

Figure S2 Accumulation of heterogeneous U14 in mutants of the Ccr4-Not complex. A. Total cellular RNAs isolated from the indicated strains were analyzed by northern blot with a probe against mature U14. The position of mature and extended and polyadenylated U14 is indicated on the right of the blot. A low exposure of the blot was added as a bottom panel to be able to assess the relative levels of mature U14. B. Total cellular RNAs isolated from the indicated strains were analyzed by northern blot with probes against several 3prime-extended snoRNAs (U18, snR71 and U3). The positions of mature and extended snoRNAs are indicated on the right of the blot.
(0.89 MB TIF)

Figure S3 Low unspecific background binding of proteins in the tandem affinity purification. Total protein extracts prepared from the indicated strains were subject to the tandem affinity purification protocol. The proteins eluted from the second column were separated by SDS-PAGE and the gel was stained with coomassie. Mass spectrometry analysis of the visible proteins did not reveal any Ccr4-Not complex or exosome subunit (data not shown).
(0.36 MB TIF)

Figure S4 Analysis of RNA in glycerol gradient fractionation of total cell extracts. RNA was extracted from 250 microlitre of the glycerol gradient fractions obtained from wild-type cells expressing Tap-tagged Rrp41 (see Fig. 4C, top panel), and these fractions were then analyzed on a 1% agarose gel which was further stained with ethidium bromide to reveal abundant RNAs. The visible tRNAs, and the 18S and 25S rRNAs are indicated.
(0.37 MB TIF)

Figure S5 Not5 co-immunoprecipitates with subunits of the exosome and TRAMP complexes. Total protein extracts were prepared from wild-type cells expressing Tap-tagged Rrp45, Rrp46, Mtr3, Rrp43, Rrp40, Rrp4 or Air2, namely from strains MY6425, MY6430, MY6428, MY6429, MY6423, MY5567 or MY7010. 2mg of total protein extracts was incubated with (IP) or without (IP0) antibodies against CBP. 50 mg of total extract (TE), equivalent volume of unbound extract (FT) and the immunoprecipitate were loaded on SDS-PAGE followed by western blotting with antibodies against Not5, which revealed both Not5 and the Tap-tagged proteins as indicated.
(0.49 MB TIF)

Figure S6 RNA is required for the integrity of the largest Rrp41-containing complexes. 5 mg of total protein extracts prepared from wild-type cells expressing Tap-tagged Rrp41 and treated or not with RNAse A as indicated were loaded on a glycerol gradient. Proteins in the different fractions of the gradient were precipitated by TCA and analyzed by SDS-PAGE and western blotting for the presence of Tap-tagged Rrp41 with antibodies against CBP. This gradient was spun for 12 rather than 10 hours leading to greater sedimentation. Hence the first peak of Rrp41 sediments in fractions 10–13 rather than fractions 3–8 as in Fig. 4.
(0.25 MB TIF)

Figure S7 Verification of RNA digestion for total cellular extracts treated with RNAse A. The RNA present in the total protein extracts prepared from not4delta or caf40delta cells expressing tagged Not4 or tagged Caf40 respectively, that were digested or not with RNAse A (see Fig. 7), was analyzed by migration on a 1% agarose gel stained with ethidium bromide.
(0.29 MB TIF)

Table S1 The results of the micro-array analyses for all of the snRNAs and snoRNAs present on the ChIPs are indicated, as well as a description of the types of snoRNAs as obtained from the yeast snoRNA database at US Amherst (http://people.biochem.umass.edu/fournierlab/snornadb/main.php).
(0.03 MB XLS)

Table S2 List of the peptides identified by mass spectrometry with scores for identified proteins.
(0.03 MB XLS)

Table S3 List of the peptides identified by mass spectrometry with scores for identified proteins.
(0.03 MB XLS)
