# Supplementary figures and images for: The CCR4-NOT Complex Physically and Functionally Interacts with TRAMP and the Nuclear Exosome
Source: PLoS One. 2009 Aug 25;4(8):e6760. doi: 10.1371/journal.pone.0006760 (PMC2727002; doi:10.1371/journal.pone.0006760)

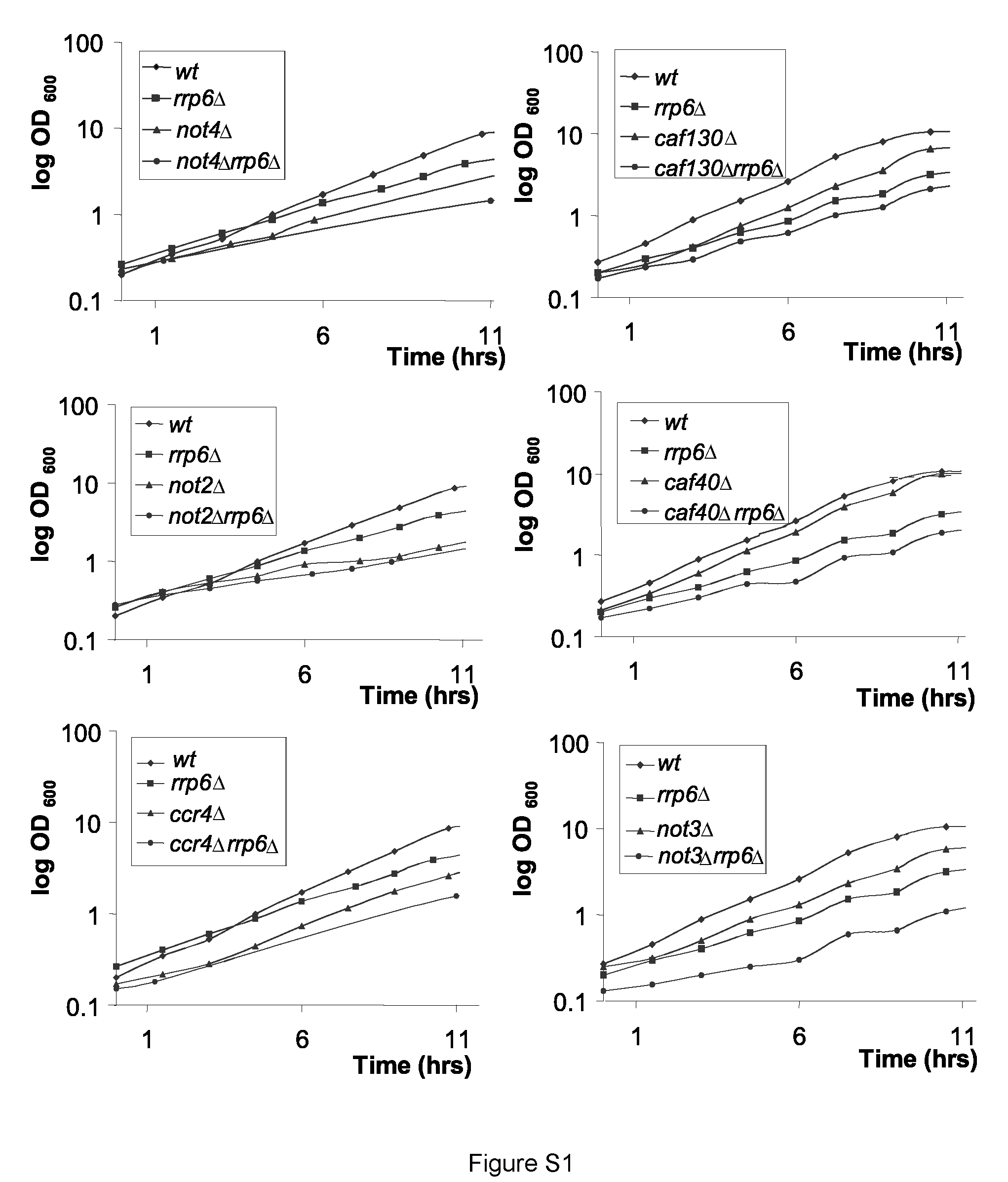

Supplement: Figure S1 — Synthetic growth phenotypes when deletions of RRP6 and the Ccr4-Not complex are combined. The indicated strains were grown at 30°C exponentially in high glucose for 24 hours, then diluted to an OD600 of 0.2 and followed for growth during the next 11 hours by measuring the OD600. (0.23 MB TIF) [file pone.0006760.s001.tif]

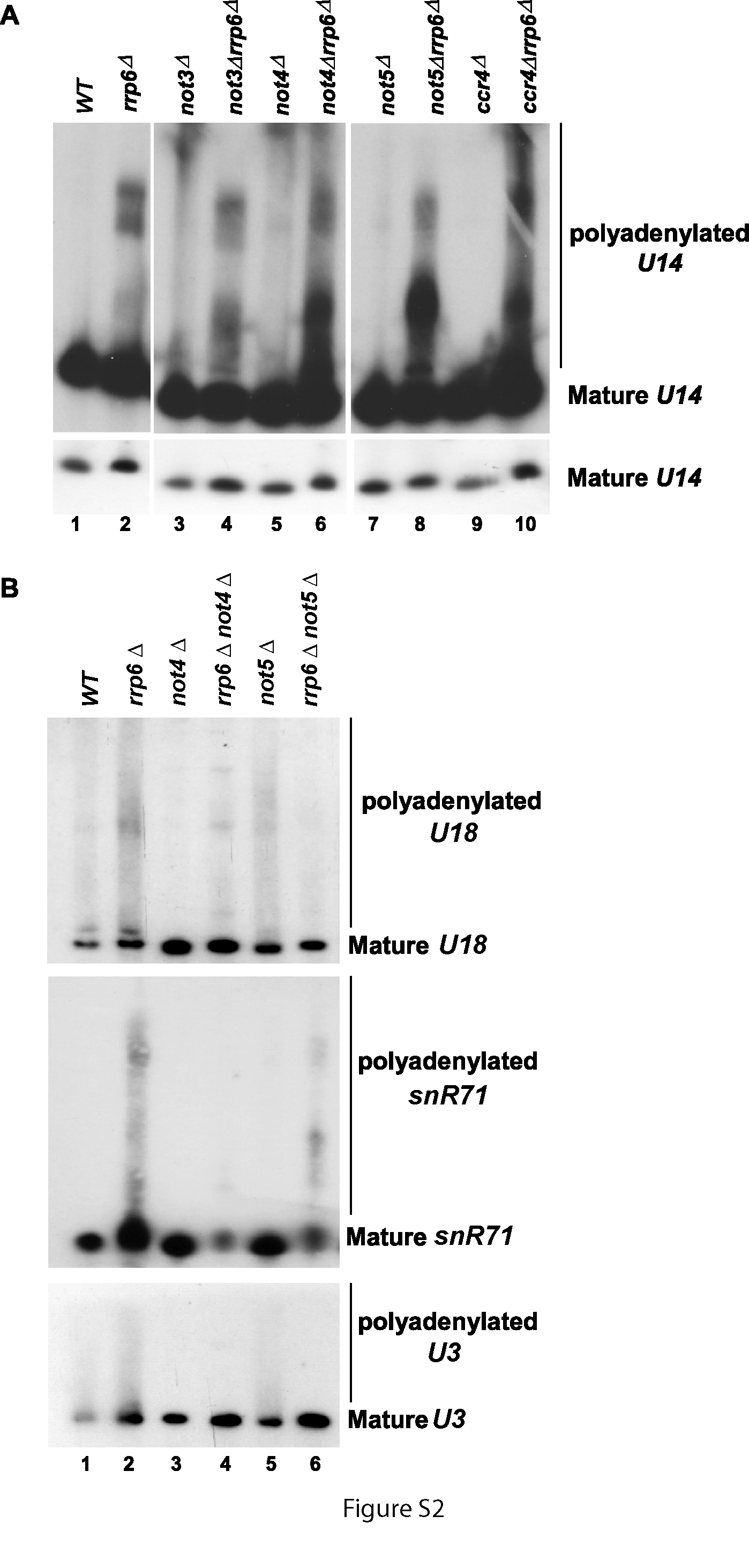

Supplement: Figure S2 — Accumulation of heterogeneous U14 in mutants of the Ccr4-Not complex. A. Total cellular RNAs isolated from the indicated strains were analyzed by northern blot with a probe against mature U14. The position of mature and extended and polyadenylated U14 is indicated on the right of the blot. A low exposure of the blot was added as a bottom panel to be able to assess the relative levels of mature U14. B. Total cellular RNAs isolated from the indicated strains were analyzed by northern blot with probes against several 3prime-extended snoRNAs (U18, snR71 and U3). The positions of mature and extended snoRNAs are indicated on the right of the blot. (0.89 MB TIF) [file pone.0006760.s002.tif]

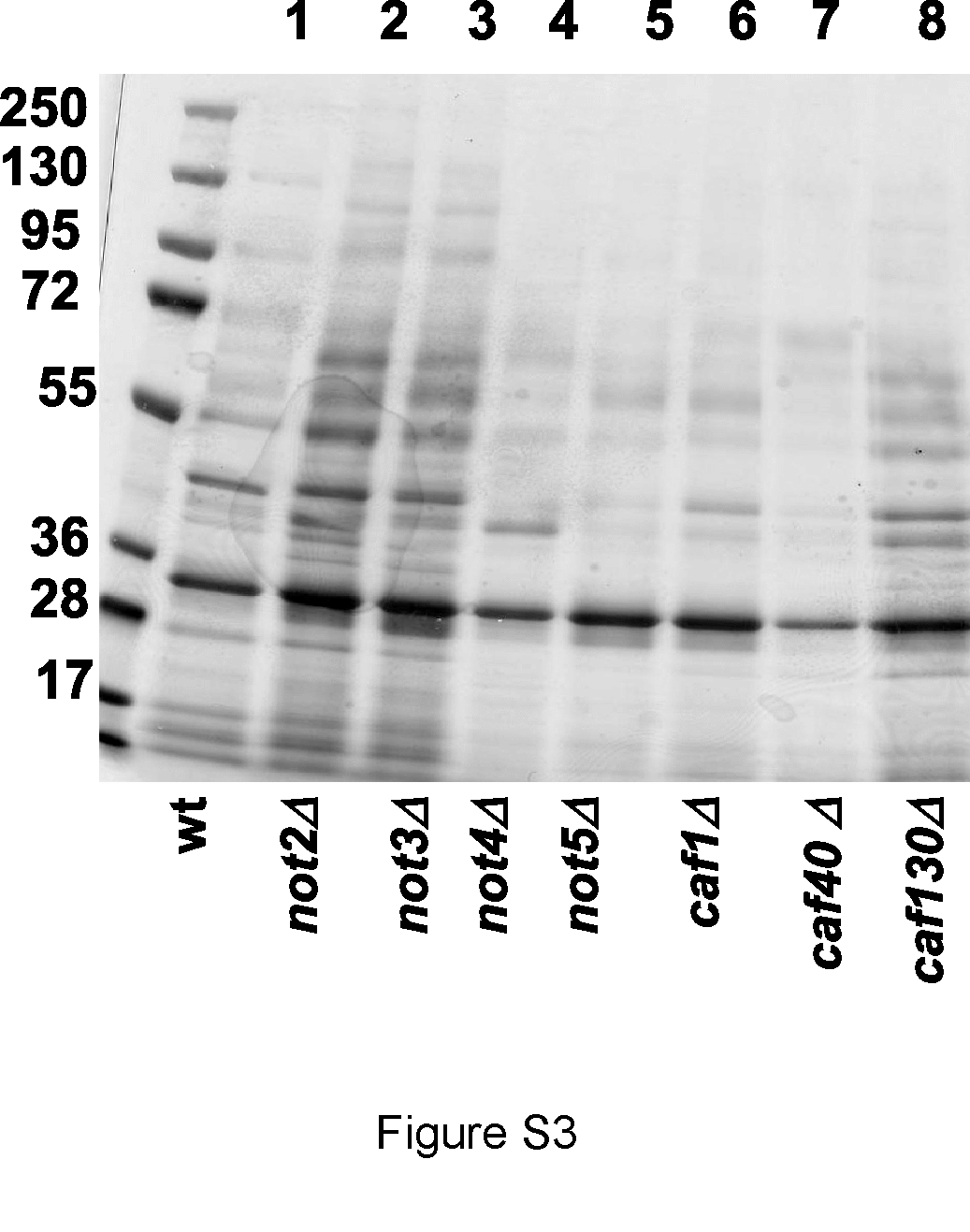

Supplement: Figure S3 — Low unspecific background binding of proteins in the tandem affinity purification. Total protein extracts prepared from the indicated strains were subject to the tandem affinity purification protocol. The proteins eluted from the second column were separated by SDS-PAGE and the gel was stained with coomassie. Mass spectrometry analysis of the visible proteins did not reveal any Ccr4-Not complex or exosome subunit (data not shown). (0.36 MB TIF) [file pone.0006760.s003.tif]

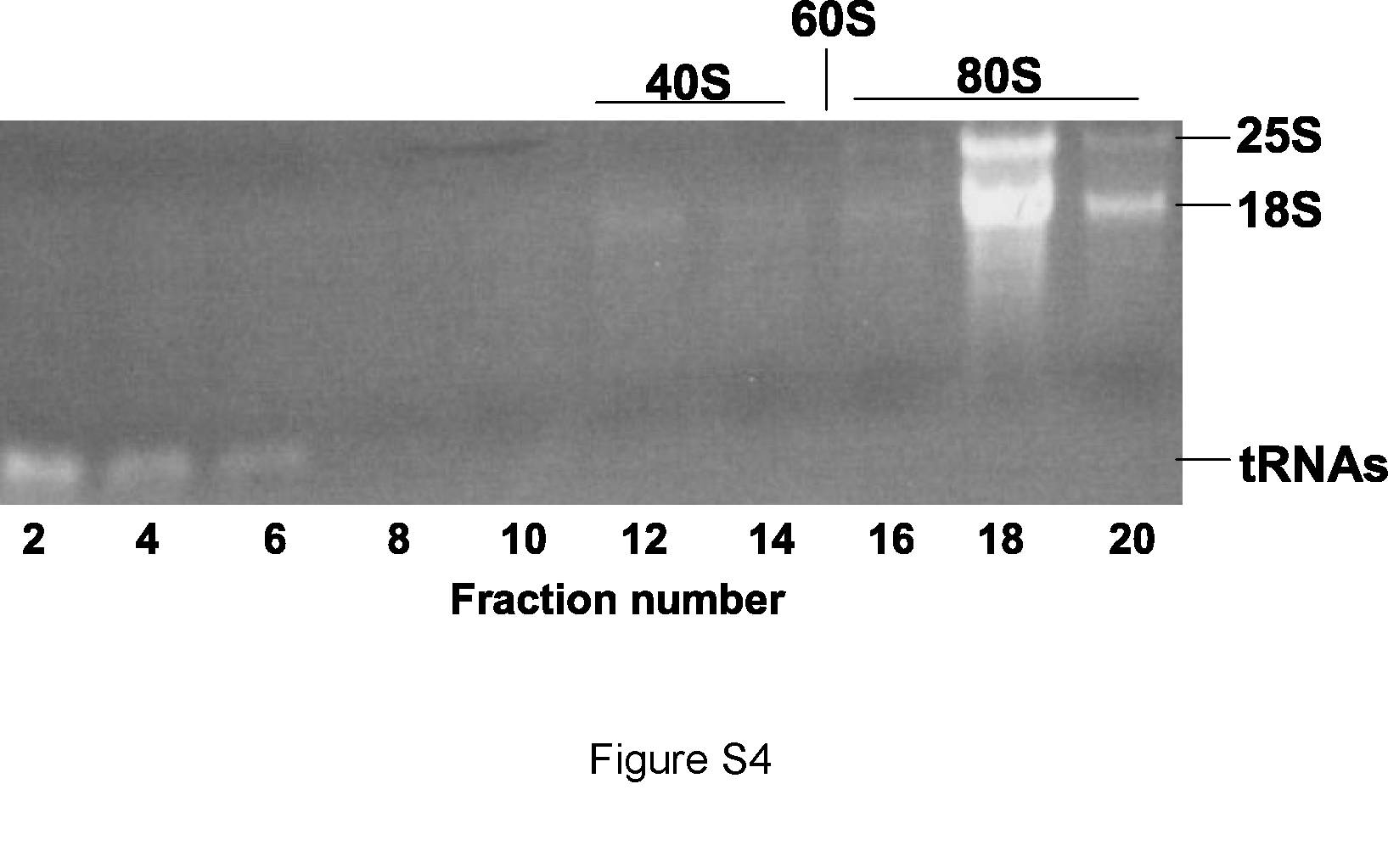

Supplement: Figure S4 — Analysis of RNA in glycerol gradient fractionation of total cell extracts. RNA was extracted from 250 microlitre of the glycerol gradient fractions obtained from wild-type cells expressing Tap-tagged Rrp41 (see Fig. 4C, top panel), and these fractions were then analyzed on a 1% agarose gel which was further stained with ethidium bromide to reveal abundant RNAs. The visible tRNAs, and the 18S and 25S rRNAs are indicated. (0.37 MB TIF) [file pone.0006760.s004.tif]

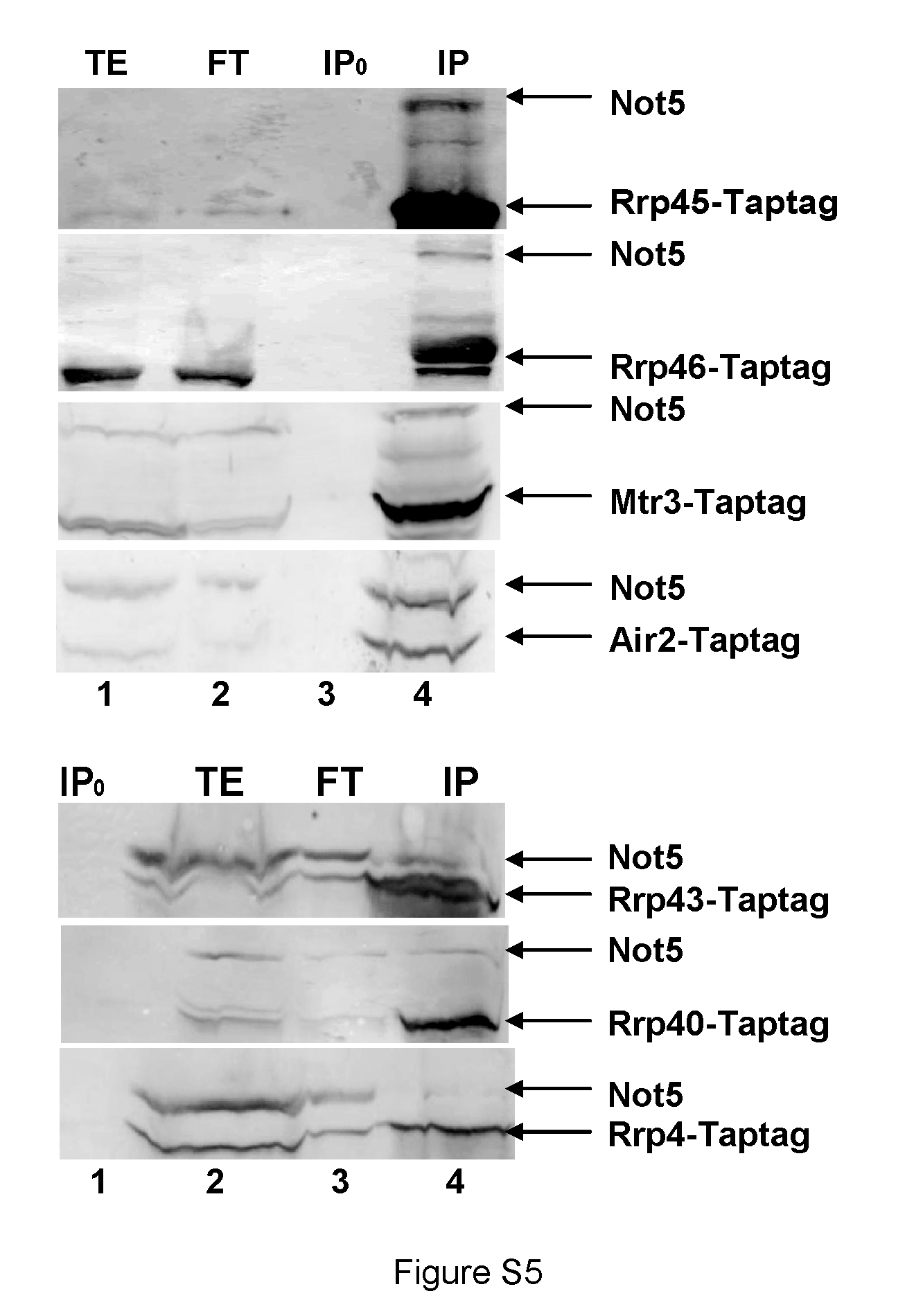

Supplement: Figure S5 — Not5 co-immunoprecipitates with subunits of the exosome and TRAMP complexes. Total protein extracts were prepared from wild-type cells expressing Tap-tagged Rrp45, Rrp46, Mtr3, Rrp43, Rrp40, Rrp4 or Air2, namely from strains MY6425, MY6430, MY6428, MY6429, MY6423, MY5567 or MY7010. 2mg of total protein extracts was incubated with (IP) or without (IP0) antibodies against CBP. 50 mg of total extract (TE), equivalent volume of unbound extract (FT) and the immunoprecipitate were loaded on SDS-PAGE followed by western blotting with antibodies against Not5, which revealed both Not5 and the Tap-tagged proteins as indicated. (0.49 MB TIF) [file pone.0006760.s005.tif]

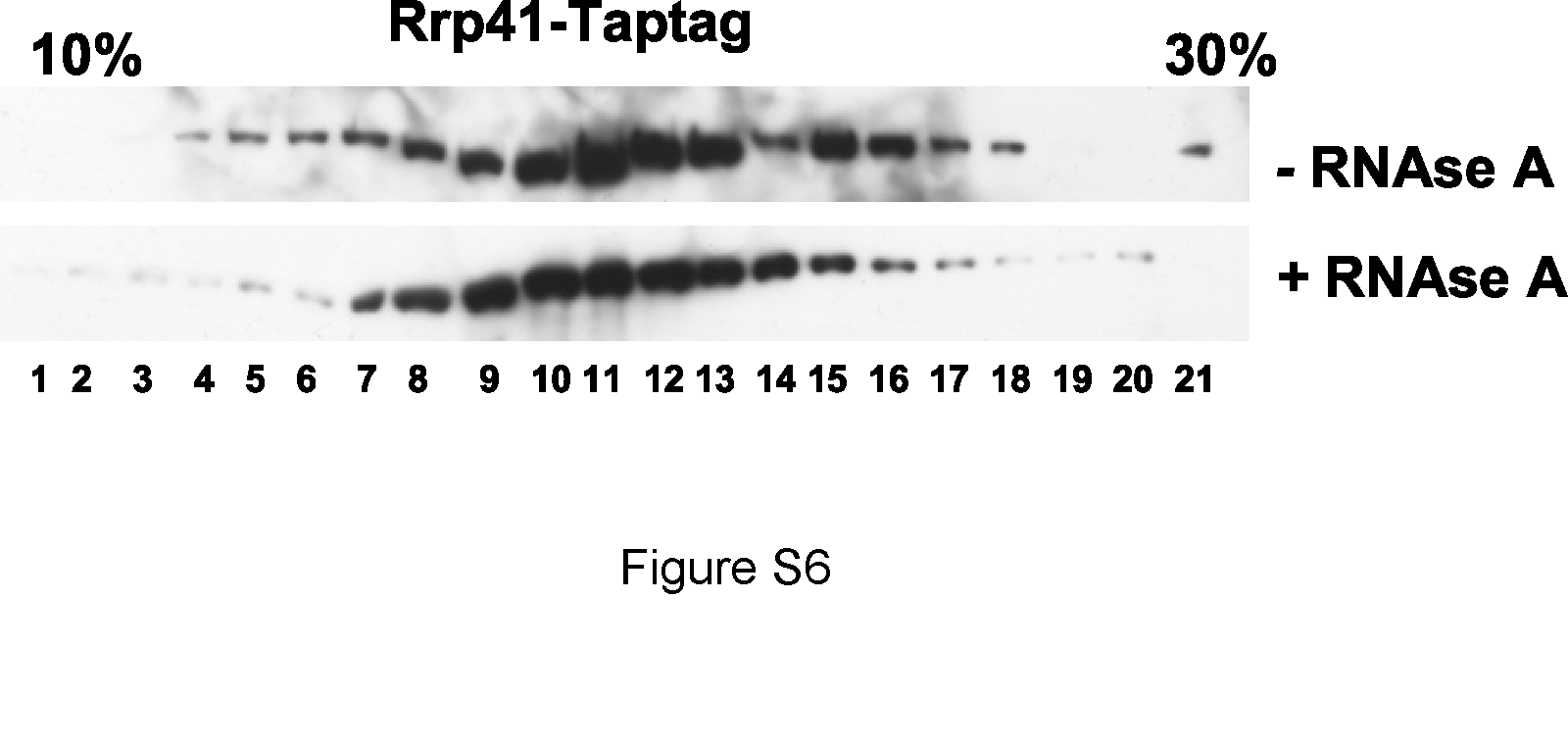

Supplement: Figure S6 — RNA is required for the integrity of the largest Rrp41-containing complexes. 5 mg of total protein extracts prepared from wild-type cells expressing Tap-tagged Rrp41 and treated or not with RNAse A as indicated were loaded on a glycerol gradient. Proteins in the different fractions of the gradient were precipitated by TCA and analyzed by SDS-PAGE and western blotting for the presence of Tap-tagged Rrp41 with antibodies against CBP. This gradient was spun for 12 rather than 10 hours leading to greater sedimentation. Hence the first peak of Rrp41 sediments in fractions 10–13 rather than fractions 3–8 as in Fig. 4. (0.25 MB TIF) [file pone.0006760.s006.tif]

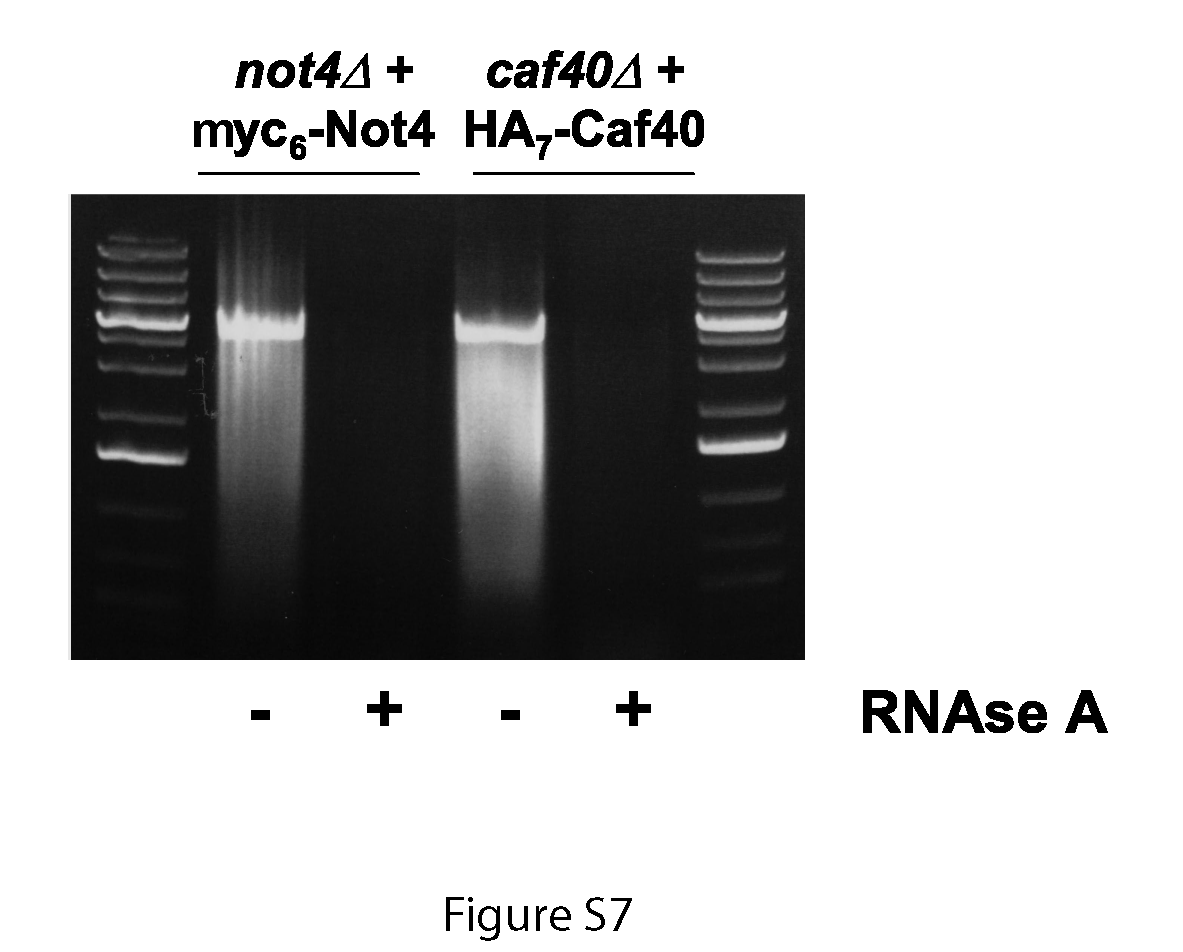

Supplement: Figure S7 — Verification of RNA digestion for total cellular extracts treated with RNAse A. The RNA present in the total protein extracts prepared from not4delta or caf40delta cells expressing tagged Not4 or tagged Caf40 respectively, that were digested or not with RNAse A (see Fig. 7), was analyzed by migration on a 1% agarose gel stained with ethidium bromide. (0.29 MB TIF) [file pone.0006760.s007.tif]
